# A244 EVIDENCE THAT IMPAIRED VAGAL SATIETY SENSING IN OBESITY IS DRIVEN BY LUMINAL MEDIATORS

**DOI:** 10.1093/jcag/gwae059.244

**Published:** 2025-02-10

**Authors:** A Chakraborty, S Sachdev, D E Reed, A E Lomax

**Affiliations:** Biomedical and Molecular Sciences, Queen’s University, Kingston, ON, Canada; Biomedical and Molecular Sciences, Queen’s University, Kingston, ON, Canada; Biomedical and Molecular Sciences, Queen’s University, Kingston, ON, Canada; Biomedical and Molecular Sciences, Queen’s University, Kingston, ON, Canada

## Abstract

**Background:**

Obesity occurs when energy intake exceeds energy expenditure. The physiological changes causing excess energy intake are unclear. Satiety is conveyed from the gut to the brain by vagal afferent neurons (VANs) that have cell bodies in the nodose (NG) and axons that connect the gut and brain stem. There is evidence of diminished satiety sensation in obesity. In the current study, we characterised VAN activity in the jejunum of obese mice and investigated whether mediators secreted into the lumen contribute to this effect. We hypothesized that obese mice would have diminished VAN activity and secrete luminal mediators that inhibit VAN signalling.

**Aims:**

Characterise changes in VAN satiety sensing in obese mice and determine if luminal mediators can induce these changes.

**Methods:**

Genetically leptin-deficient male [N = 6] and female obese (Ob) [N = 8] mice were used, with heterozygous littermates (Het) [male N = 7, female N = 7] serving as controls. *Ex-vivo* afferent nerve recordings were conducted on VAN axons attached to the mouse jejunum. Changes in firing frequency were assessed in response to meal-related hormonal and mechanical stimuli, namely 100 nM cholecystokinin (CCK) and distension to 60 mmHg. Additionally, naïve NG neurons were incubated in luminal contents collected from the small intestine of Ob and Het mice to determine the potential role of luminal mediators. Patch clamp recordings were carried out on these cells and changes in excitability were assessed using rheobase (higher rheobase indicates decreased excitability).

**Results:**

Ob mice had rates of basal jejunal afferent discharge 70% lower than Het mice (p <0.01) and showed 60% less activation in response to 100 nM CCK (p <0.05). Ob mice also showed a 40% attenuation in distension response compared to Het mice (p <0.01). In patch clamp experiments, NG cells from Ob mice (n = 23 cells from N = 8 mice) had a 33% higher rheobase than Het (n = 18 cells from N = 8 mice; p <0.01). Overnight incubation of NG neurons from naïve mice with luminal contents collected from the jejunum of Ob mice increased the rheobase to over double the rheobase of cells incubated with samples from Het control mice (Ob n = 12, het n = 12, p <0.05). These changes to rheobase were blocked by pre-incubation of NG neurons with the orexin-1 receptor agonist SB-334867 (p <0.01).

**Conclusions:**

Our findings suggest that during obesity, mediators are produced that can attenuate vagal satiety signaling. The ability to inhibit these changes using the orexin-1 receptor antagonist implicate orexin receptors in impaired vagal satiety signaling during obesity.

**Funding Agencies:**

None

